# Morphological, ultrastructural, genetic characteristics and remarkably low prevalence of macroscopic *Sarcocystis* species isolated from sheep and goats in Kurdistan region, Iraq

**DOI:** 10.3389/fvets.2023.1225796

**Published:** 2023-09-28

**Authors:** Salh Nawshirwan, Nicole Heucken, Nadin Piekarek, Tim van Beers, Nicole Fulgham-Scott, Andrea Grandoch, Wolfram F. Neiss, Johannes Vogt, Mohammed Barham

**Affiliations:** ^1^Directorate of Veterinary Medicine, Sulaymaniyah, Iraq; ^2^Department II of Anatomy, Faculty of Medicine, University Hospital Cologne, Cologne, Germany; ^3^Experimental Medicine, Faculty of Medicine, University Hospital Cologne, Cologne, Germany; ^4^Department I of Anatomy, Faculty of Medicine, University Hospital Cologne, Cologne, Germany; ^5^Department for Oral and Craniomaxillofacial and Plastic Surgery, Faculty of Medicine, University of Cologne and University Hospital of Cologne, Cologne, Germany; ^6^Cluster of Excellence for Aging Research (CECAD) and Center of Molecular Medicine Cologne (CMMC), University of Cologne, Cologne, Germany

**Keywords:** genetic analysis, goat, sheep, *Sarcocystis gigantea*, *Sarcocystis medusiformis*, *Sarcocystis moulei*

## Abstract

**Introduction:**

*Sarcocystis* is a genus of cyst-forming parasites that infest both humans and livestock. Some parasites cause clinical and subclinical diseases in their hosts, resulting in economic losses.

**Methods:**

Esophagus, diaphragm, and skeletal muscle from slaughtered sheep and goats were examined macroscopically, microscopically, and ultrastructurally and subjected to DNA analysis.

**Results:**

We isolated macrocysts of *S. gigantea* and of *S. caprafelis* moulei from naturally infected sheep (Ovis aries) and goats (Capra hircus). The macrocyst wall thickness was 18.9 µm in sheep and 15.3 µm in goats, and consisted of an inner Periodic acid Schiff- (PAS) negative primary wall and an outer glycoconjugates containing i.e. PAS-positive secondary wall. The walls inner surface was compartmentalized and filled with bradyzoites. In *S. gigantea* the bradyzoites were approximently 12.3 x 2.6 µm in size, while in *S. caprafelis* moulei they were 13.9 x 4.4 µm. Ultrastructurally, both species have nearly identical morphology: cauliflower-like protrusions with numerous microtubules and often dendritic-like filaments, branching from the primary wall. The 18S rRNA gene in *S. gigantea* was 85.9% identical to that in *S. medusiformis* and 80.4% to the *S. caprafelis* moulei gene. The 28S rRNA gene in *S. gigantea* was 94.6% identical to that in *S. medusiformis* and 97.3% to the *S. caprafelis* moulei.

**Conclusion:**

This study is the first to (i) detail the ultrastructure of the macrocyst wall of *S. caprafelis moulei*, (ii) identify *S. medusiformis* in Iraqi sheep, and (iii) compare the prevalence of macroscopic Sarcocystis at different time periods within the same region. A positive finding was the reduction of macroscopic sarcocystosis occurrences (0.01% in sheep and 0.02% in goats) compared to our previous data from 1992 (4.1%: sheep, 33.6%: goats).

## 1. Introduction

*Sarcocystis* species are coccidian parasites, belonging to the phylum: Apicomplexa, class: Conoidasida, order: Eucoccidiorida, and family: Sarcocystidae (1). They are distributed worldwide and parasitic to a broad range of vertebrates, such as cattle, sheep and goats. *Sarcocystis* spp. have also been isolated in Europe: Germany (2) Lithuania (3), Italy (4), the Netherlands (5) and Spain (6). Sheep are intermediate hosts for at least four species of *Sarcocystis*: *S. gigantea* (syn. *S. ovifelis*), *S. medusiformis*, *S. tenella* and *S.arieticanis*. Both species, *S. gigantea* and *S. medusiformis* are transmitted through felids and produce macrocysts. *S. tenella* and *S. arieticanis,* on the other hand, are transmitted through canids and produce microcysts (1, 7, 8). Infections from *S. tenella*, *S. gigantea* and *S. arieticanis* are common worldwide, whereas *S. medusiformis* infections have been exclusively reported in Australia, New Zealand, Iran and Italy (4, 9).

Goats are intermediate hosts for at least three *Sarcocystis* species: *S. capracanis*, *S. hircicanis*, and *S. caprafelis moulei*. *S. capracanis* and *S. hircicanis* are transmitted through canids [review (1)], and generally produce microcysts. On the other hand, *S. caprafelis moulei* [termed *S. moulei* (10–12)] is transmitted by felids and produces macrocysts (1).

The prevalency of macroscopic sarcocystosis in domestic sheep varied regionally: 4.1% in Iraq (13), 29.3% in Iran (14), and 42.7% in Egypt (15). In domestic goats macrocysts occurred 33.6% in Kurdistan/Iraq (11), 16.6% in Iran (16) and 35.5% in Egypt (15). There is but a single, brief description of the *S. moulei* macrocysts ultrastructure in Egyptian goats (17). In comparison, many studies described the localization, the pathogenesis, the cyst wall and bradyzoites structure and ultrastructure, as well as the *S. gigantea* DNA analysis in both natural and experimentally-infected sheep (1, 13, 18).

This study aims to identify *Sarcocystis* species isolated from muscle macrocysts from naturally infected domestic sheep and goats. In addition, we compare the prevalence of macroscopic sarcocystosis in the same region at different time periods. Finally, we demonstrate the differences in macro- and microscopic structure, ultrastructural morphology, and genetic characterization of macrocysts between *S. gigantea* and *S. caprafelis moulei.* To our knowledge, this is the first study (i) to describe the macrocyst wall structure using PAS-staining, (ii) to study in detail the ultrastructure of macrocysts, including protrusions and filaments of *S. caprafelis moulei* in goats, and (iii) to demonstrate isolates of *S. medusiformis* in domestic sheep in Iraq.

## 2. Materials and methods

### 2.1. Animals

A total of 141,260 domestic sheep (*Ovis aries*; 98,638 male and 42,622 female) and 37,399 domestic goats (*Capra hircus*; 32,441 male and 4,958 female) were slaughtered and investigated between September 2021 and March 2022 in 6 regional abattoirs (Sulaimany, Piramagroon, Khalakan, Darbandixan, Takya and Soran) in the northern Iraq Kurdistan region (Supplementary material S1). All slaughtered animals were carefully inspected by specialist personal and in case of any pathological changes reinspected by veterinarians. If *Sarcocystis* macrocysts were detected in esophagus, diaphragm or skeletal muscles, all obvious macrocysts and the surrounding muscle tissue were collected, and age (as estimated by dental examination) and sex of the respective animal were recorded. All tissue sampling was performed under the supervision and with permission of the state veterinarian in the above-mentioned slaughterhouses. Written consent from the animal’s owners for the participation of their animals in this study was not required in accordance with the national legislation and the institutional requirements.

### 2.2. *Sarcocystis* macrocysts

Out of a total of 141,260 sheep, only 20 had *Sarcocystis* macrocysts, and out of a total of 37,399 goats only nine carried *Sarcocystis* macrocysts, all of which were embedded in the muscular tissue and were milky-white and grainy in appearance. All 29 infected animals appeared healthy and showed no signs of disease before slaughter. Of the 20 infected sheep, we collected 21 pieces of infected tissue: 11 from skeletal muscle, five from the diaphragm, and five from the esophagus, namely one sheep had macrocysts in both skeletal muscle and esophagus. Of the nine infected goats, 12 tissue pieces were collected, nine from the esophagus and three from skeletal muscle. Here, three goats exhibited macrocysts in both the esophagus and skeletal muscle. Each tissue piece contained a minimum of two and a maximum of 12 *Sarcocystis* macrocysts. The size of *Sarcocystis* macrocysts in sheep and goats were then measured using a digital caliper. Tissue pieces from esophagus and skeletal muscle containing macrocysts were randomly divided into three groups, and then immediately fixated by immersion in the following solutions: 4% paraformaldehyde (PFA) in 0.1 M phosphate butter pH 7.4 for histological examination, 70% ethanol for DNA purification, and sequencing or 2% glutaraldehyde (GLA) plus 2% PFA in 0.1 M phosphate butter pH 7.4 for transmission electron microscopy.

### 2.3. Histological examination

Ten of the 21 macrocysts-containing sheep samples, and four of the 12 macrocysts-containing goat samples were fixed in 4% buffered formaldehyde, embedded in paraffin, serially sectioned at 5 μm thickness with a rotary microtome (Leica Autocut 1,140). The slides containing tissue sections were then stained with Tri-PAS (30 min 1% Periodic acid at room temperature 10 min Schiffś reagent (19) Mayer’s hematoxylin and methylene blue. All samples were further imaged with a Zeiss AX10 light microscope and a Hitachi HV-F202 camera. The following measurements were all measured using image analysis software Image-Pro® Plus version 6.0 (Media Cybernetics, Inc. MD 20910, United States): (i) thickness of the degenerating layer, primary and secondary cyst walls, villous protrusions, and internal cyst compartments, and (ii) bradyzoites dimensions.

### 2.4. Transmission electron microscopy

Twelve *Sarcocystis* macrocysts from sheep and four from goats were collected from the esophagus and skeletal muscles and immersion-fixed at 4°C in a solution containing 2% GLA plus 2% PFA in 0.1 M Phosphate buffer, pH 7.4, and left in solution for up to 2 weeks for logistical reasons. After nine rinses in 0.1 M cacodylate buffer pH 7.4, the cysts were postfixed for 2 h with 1% OsO4 + 1.5% K3Fe^3^ (CN) 6 in 0.1 M cacodylate buffer, pH 7.4 (20, 21). The tissues were dehydrated *via* acetone/propylene oxide and embedded in epoxide resin (epon; Fluka, Switzerland).

For morphometric light microscopical analysis, 0.5 μm thick cross sections of epoxide embedded cysts were stained with methylene blue. Ultrathin sections (30 nm) were cut with a diamond knife, mounted on formvar/carbon-coated 200-mesh copper grids and contrasted with uranyl acetate and lead citrate solution. TEM was performed with a Zeiss EM109 (80 kV, 200 μm condenser and 30 μm objective apertures, TRS-2 K-Camera). Magnification of micrographs was calibrated by means of a cross-grating replica (2,160 lines/mm; Polaron, England).

To analyze morphology, methylene blue stained semithin sections of several macrocysts were imaged with a Zeiss AX10 light microscope. For the investigation of cyst wall morphology, villous protrusions, and bradyzoites, the length and width of these *Sarcocystis* components were measured using the same image analysis software as for paraffin sections (see above).

### 2.5. DNA purification

The collected macrocysts from sheep (*n* = 10) and goats (*n* = 4) were stored in 70% ethanol until DNA extraction. The samples were briefly lysed in lysis buffer (200 mM Tris pH8, 50 mM EDTA pH8; 100 mM NaCl, 1% SDS in H2O), supplemented with 30 U/mg Proteinase K at 600 rpm and 55°C overnight. The following day, samples were centrifuged 8 min at 14000 rpm to separate the genomic DNA in the supernatant from cell debris in the pellet. The supernatant was transferred to fresh tubes containing 500 μL ice cold 100% EtOH resulting in DNA filaments to form. For washing, the DNA filaments were collected with a Pasteur pipette tip from the liquid and transferred into a new tube containing 375 μL ice cold 70% EtOH. Finally, DNA was resuspended in 75 μL ultra pure nuclease-free water overnight at 42°C and stored at 4°C until further analysis.

### 2.6. PCR amplification, gel extraction and DNA sequencing

New primers for amplification of the 18S rRNA and 28S rRNA genes were designed, so that one primer pair each could be used to amplify the gene of interest from all of the mentioned species (*S. gigantea*, *S. medusiformis* and *S. caprafelis moulei*). Therefore, the reference sequences for either the 18S rRNA or 28S rRNA genes of *S. gigantea*, *S. medusiformis* and *S. caprafelis* were aligned. Primer binding sites were selected in sections were all three reference sequences have 100% sequence identity and surrounding sections where the sequences had the greatest differences between the three named species. The primer pair oBM001 (5′- ACTGCGAATGGCTC ATTAAAACA-3′), and oBM002 (5’TGATCGTCTTCGAGCCCCTA- 3′) amplified a fragment between 1,001 bp and 1,072 bp from the 18S rRNA gene of each species. For the 28S rRNA, the primers oBM003 (5′-AGCGGTGGAGAAGAAAATAACA-3′) and oBM004 (5′-TCACATGGAACCCTTCTCCA-3′) resulted in a fragment of 1,674 bp, 1,676 bp, and 1,680 bp, respectively.

PCR amplification was performed in a thermocycler using the following program for 18S rRNA: Initial denaturation at 95°C (3 min), followed by 30 cycles of 95°C (30s), 60°C (30s) and 72°C (70s), ending with a final elongation at 72°C (5 min). For the 28S rRNA, the program differed slightly: Initial denaturation at 95°C (3 min), followed by 30 cycles of 95°C (30s), 58°C (30s) 72°C (1 m and 45 s), ending with a final elongation at 72°C (5 min).

PCR products were run on a 1% agarose gel together with GeneRuler 100 bp and 1 kb plus DNA ladders (Thermofischer), stained with 0,1 μg/mL Ethidiumbromide, and further analyzed after electrophoresis with a UV transilluminator. Proportionally correct bands were excised from the gel and underwent DNA extraction using the NucleoSpin Gel and PCR Clean-up kit (Macherey-Nagel), according to the manufacturer’s instructions.

For each Cyst, one PCR product per gene of interest was sequenced with forward and reverse primers for the respective gene in two individual reactions.

Sequencing results were obtained from Microsynth Seqlab^1^ and analyzed in SnapGene and with the NCBI Blast Tool.^2^

### 2.7. Sequence databases

Nucleotide sequences referred to as reference sequences or BLAST results mentioned in this paper are available in the GenBank™ NCBI nucleotide databases under the accession numbers: MK420020.1, MK420025.1, MK420021.1, MK420026.1, L76473.1, AF012884.1, KP053891.1, MK045326.1, and GQ131808.1. All our data sequences for 18S rRNA and 28S rRNA genes are deposited in NCBI under the accession numbers (see Supplementary material S1).

### 2.8. Statistical evaluation

All values (dimentions of macrocysts, bradyzoites and villar protrusions, thickness of cyst walls and degenerating layers) are expressed as the mean ± s.e.m. (standard error of the mean). Following outlier analysis, two group comparisons were performed using an unpaired two-tailed *t*-test for normal distributed data and a Mann–Whitney test for nonparametric data. Corresponding details are given in the figure legend. Analysis were performed using GraphPad Prism 9.2.0. Statistical significance was considered at *p* < 0.05 and marked with *, *p* < 0.01 was assigned with **, *p* < 0.001 with ***, and *p* < 0.0001 with ****.

## 3. Results

### 3.1. Macroscopic characterization

**Figure 1:**
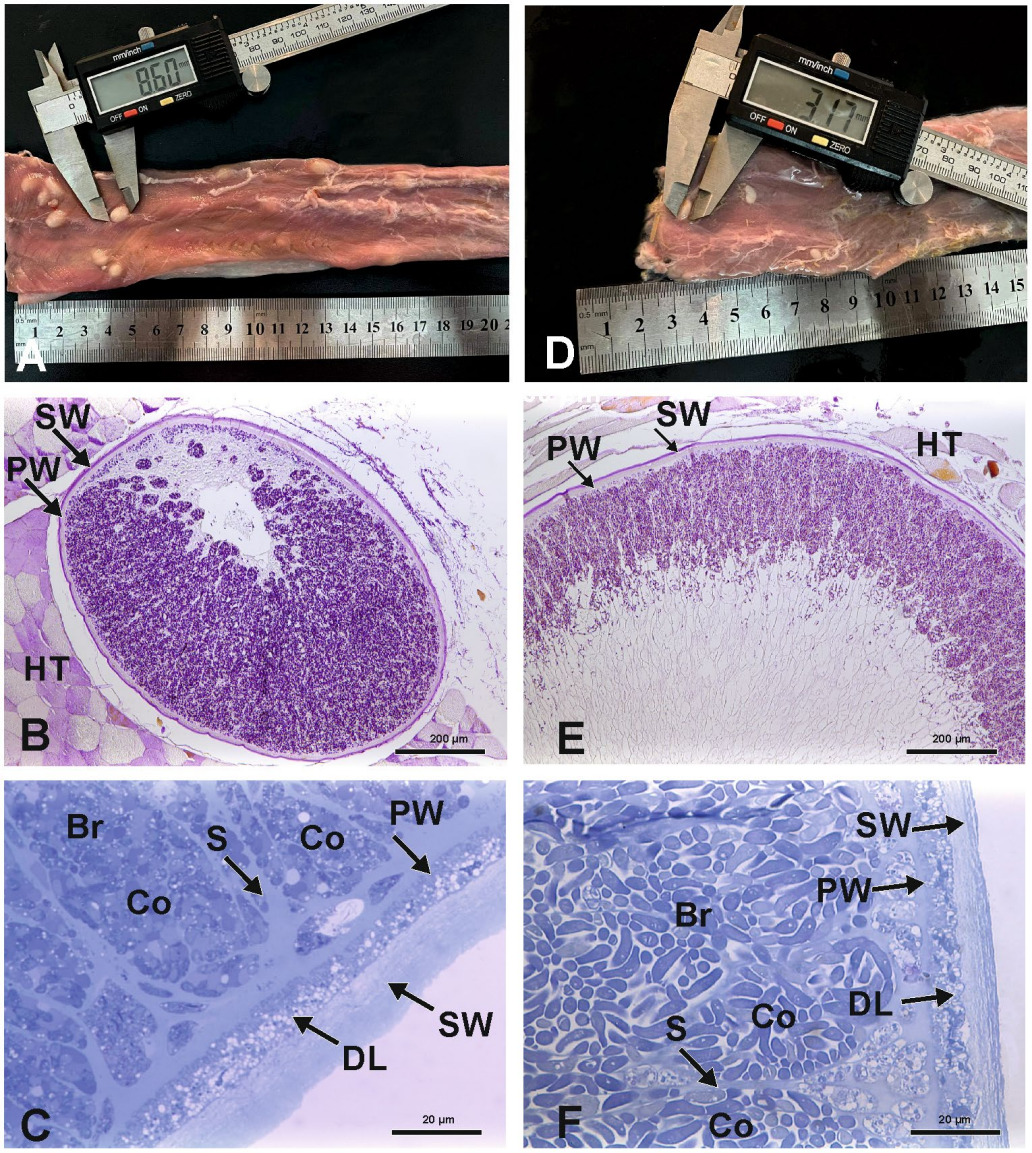
Macroscopy and light microscopy of *S. gigantea* **(A–C)** and *S. caprafelis moulei* **(D–F)** macrocysts Gross appearance of macrocysts of *S. gigantea* in sheep **(A)** and *S. caprafelis moulei* in goat **(D)** in the heavily infected esophagi, scale bar in cm. Light microscopic appearance of an oval macrocyst of *S. gigantea* (**B**) and *S. caprafelis moulei* **(E)**, both macrocysts have a PAS-positive outer secondary thick wall (SW), and a PAS-negative inner primary relatively thick wall (PW), HT = Host tissue. Higher magnification shows the primary and secondary walls of the macrocysts of *S. gigantea* **(C)** and *S. caprafelis moulei* **(F)**. Between the primary and secondary walls is a relatively thick layer of degenerating host cells (DL). Beneath the primary wall are numerous compartments (Co) separated by tissue septa (S) and filled with banana-shaped bradyzoites (Br), **(B)** and **(E)** 5 μm paraffin sections, Tri-PAS staining; **(C)** and **(F)** 0.5 μm semithin sections, methylene blue staining.

**Figure 2:**
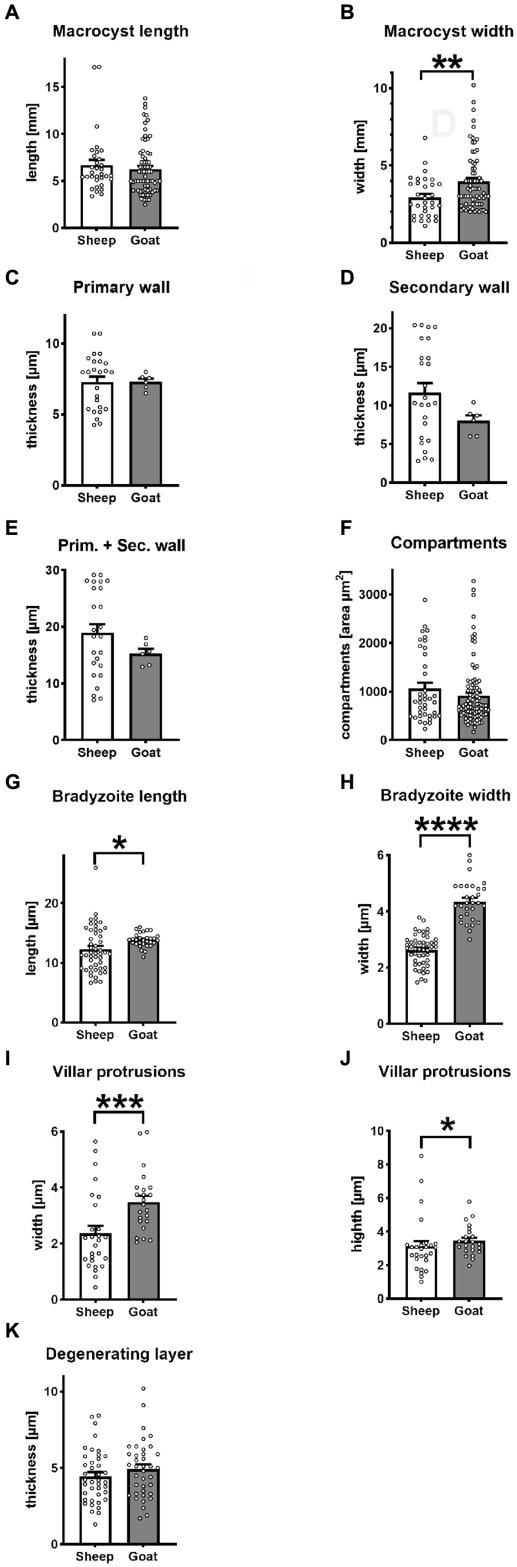
Statistical variantion of macrocyst parameters between *S. gigantea* (sheep) and *S. caprafelis moulei* (goat). In all tests *p* < 0.05 was considered significant and marked with * *p* < 0.01 was assigned with **, *p* < 0.001 with ***, and **** *p* < 0.0001. ± = standard error of the mean. **(A,B)** macrocysts length and width: *S. gigantea* 6.7× 2.9 mm, *S. caprafelis moulei* 6.3 × 4.0 mm (*n* = 32 sheep and *n* = 70 goat; two-tailed Mann–Whitney test). (**C**) Inner primary wall thickness of macrocysts: *S. gigantea* 7.3 μm, *S. caprafelis moulei* 7.3 μm. **(D)** Outer secondary wall thickness of macrocysts: *S. gigantea* 11.6 μm, *S. caprafelis moulei* 8.0 μm (*n* = 25 sheep, *n* = 6 goat; two-tailed Mann–Whitney test). **(E)** Total wall thickness of macrocysts, i.e., primary and secondary cyst walls together: *S. gigantea* 18.9, *S. caprafelis moulei* 15.3 μm (*n* = 25 sheep, *n* = 6 goat; two-tailed Mann–Whitney test). **(F)** Area compartment of macrocysts: *S. gigantea* 1,065 μm^**2**^, *S. caprafelis moulei* 917 μm^2^ (*n* = 39 sheep, *n* = 96 goat; two-tailed Mann–Whitney test). **(G)** Bradyzoite length: *S. gigantea* 12.3, *S. caprafelis moulei* 13.9 μm (*n* = 49 sheep, *n* = 30 goat; two-tailed Mann–Whitney test). **(H)** Bradyzoite width: *S. gigantea* 2.6 μm, *S. caprafelis moulei* 4.4 μm (*n* = 49 sheep, *n* = 30 goat; two-tailed t-test). **(I)** width of macrocyst villar protrusions: *S. gigantea* 2.4 μm, *S. caprafelis moulei* 3.5 μm (*n* = 27 sheep, *n* = 23 goat; two-tailed Mann–Whitney test). **(J)** height of macrocyst villar protrusions: *S. gigantea* 3.1 μm, *S. caprafelis moulei* 3.4 μm (*n* = 27 sheep, *n* = 23 goat; two-tailed Mann–Whitney test). **(K)** Thickness of the degenerating layer: *S. gigantea* 4.5 μm, *S. caprafelis moulei* 4.9 μm (*n* = 40 sheep, *n* = 38 goat; two-tailed t-test), for more details see Supplementary material S2.

Out of a total of 141,260 sheep, and 37,399 goats (Supplementary material S1) slaughtered and subsequently examined by veterinarians, only 20 sheep (0.01%) and nine goats (0.02%) contained *Sarcocystis* macrocysts. *S. gigantea* and *S. caprafelis moulei* fresh, unfixed macrocysts appeared milky-white and grainy and were always found embedded in muscle tissue. *S. gigantea* macrocysts (Figure 1) appeared large, thick, round or elongated. They were located exclusively in the diaphragm and esophagus with an average length of 6.7 mm and width of 2.9 mm. Further, *S. caprafelis moulei* macrocysts (Figure 1D; Supplementary material S2) seemed also large, thick, oval or round; they were exclusively found in the esophagus–preferentially in the lower half–with an average length of 6.3 mm and width of 4.0 mm (Figures 2A,B; Supplementary material S2). A two-tailed *t*-test revealed no significant difference (*p*<0.05) in macrocyst length, but a significant difference (*p*<0.01) in macrocyst width (Figures 2A,B) between sheep and goats. This mean difference of 2.9 mm versus 4.0 mm macrocyst width is not, however, reliably discernible by observation. The macroscopic identification of *S. gigantea* and *S. caprafelis moulei* depends solely on the source of the sample: *S. gigantea* is collected from sheep, *S. caprafelis moulei* from goats. Evidence that these truly are different macrocyst subspecies is provided only by DNA sequencing and ultrastructural analysis (see below). All structures, that were deemed macrocysts by macroscopical inspection, proved to be so by histological, electron microscopical or DNA analysis.

### 3.2. Lightmicroscopic characterization

In histological sections of muscle tissues, the macrocysts were thick-walled and consisted of two layers in both species. The mean wall thickness was 18.9 μm in *S. gigantea* and was 15.3 μm in *S. caprafelis moulei* (Figure 2E). An outer PAS-positive, i.e., covalently bound glycoconjugates containing secondary thick wall (11.6 μmin sheep; 8.0 μm in goats) was stained purple-red in Figures 1B,E, 2D. And an inner PAS-negative primary, relatively thick wall (7.3 μm in sheep and goats) was stained pale yellow in Figures 1B,E, 2C. Regarding the PAS-positive outer secondary wall, it is noteworthy that Apicomplexan protozoan parasites trigger host innate immune responses *via* their cell surface glycocojugates (22). On methylene blue staining, the secondary cyst wall appeared thick and bluish-pale in both species, whereas the primary wall was obviously thinner and purple in color. In both species, a vacuolated layer of degenerating host cells was found between the primary and secondary cyst walls (Figures 1C,F). The inner surface of the macrocysts within the primary wall was divided into a series of large round, oval or elongated compartments. The compartment sizes were approximately 1,065 μm^2^ in *S. gigantea* and 917 μm^2^ in *S. caprafelis moulei* (Figure 2F). Statistical analysis revealed no significant difference (*p* < 0.05) in the thickness of macrocyst walls and sizes of compartment between sheep and goats (Figures 2C,F). Compartments were packed with banana-shaped bradyzoites with an average size of 12.3 × 2.6 μm *in S. gigantea* (Figure 1C), and 13.9 μm´ 4.4 μm in *S. caprafelis moulei* (Figure 1F). Statistically, there is a significant difference (*p* < 0.05) in the length of bradyzoites and a highly significant difference (*p* < 0.0001) in the width of bradyzoites (Figures 2G,H) between sheep and goats. As with the macroscopical observations the differences in microscopical measurements do not allow the identification of *S. gigantea* versus *S. caprafelis moulei*, but only the source of the sample. Notably both *S. gigantea* from sheep and *S. caprafelis moulei* from goats have the same PAS-positive secondary outer cyst wall that is described here for the first time.

### 3.3. Ultrastructural characterization of macrosarcocysts

**Figure 3:**
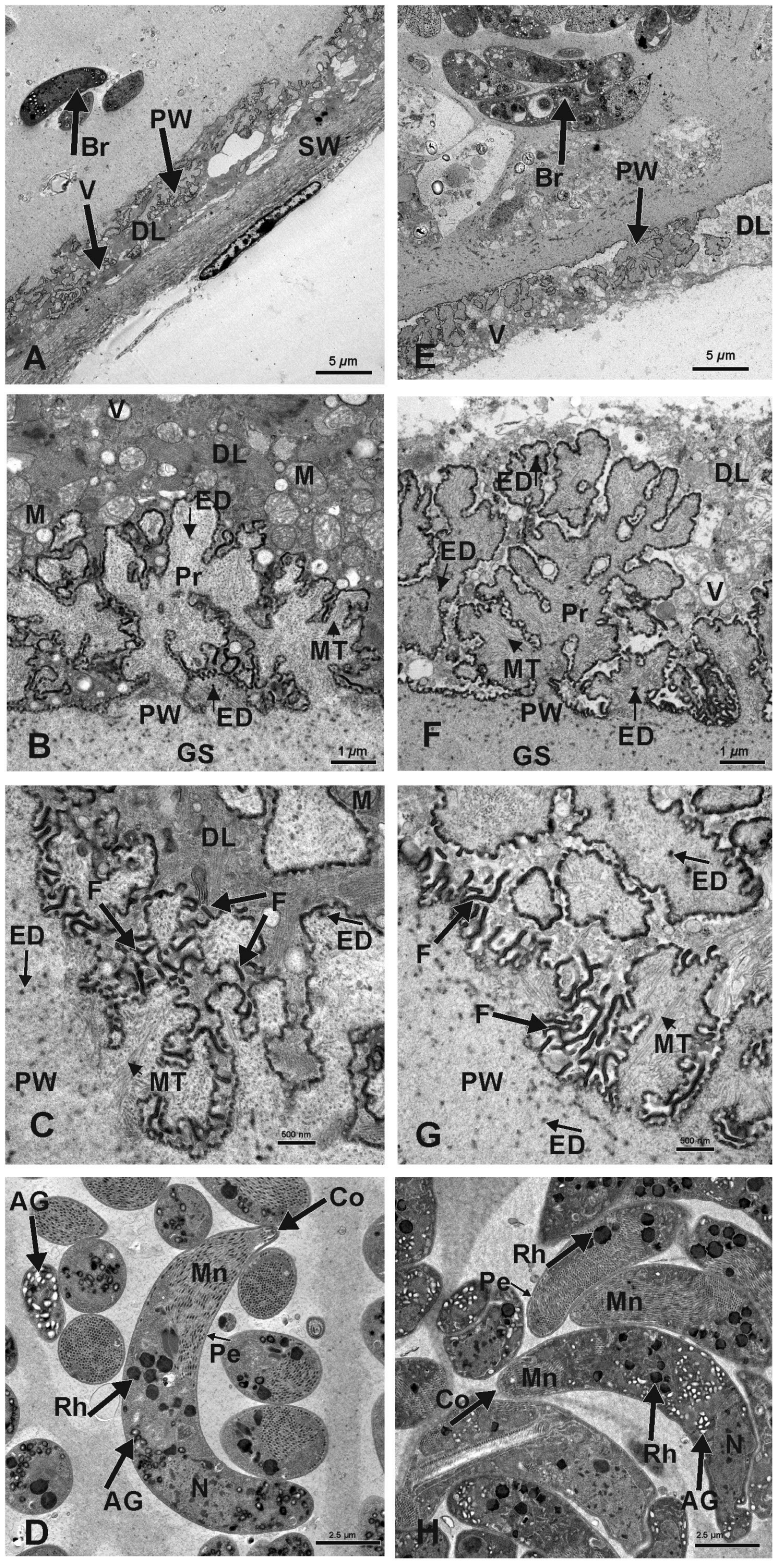
Electron micrographs of macrocysts in the esophagus of sheep (*S. gigantea*) **(A–D)** and goats (*S. caprafelis moulei*) **(E–H)**. **(A–C)** and **(E–G)**: showing the macrocyst wall of *S. gigantea* and *S. caprafelis moulei*, respectively. In both species, the degenerating host cells layer (DL) is located between the primary (PW) and secondary (SW) cyst walls and contains many vacuoles (V). In **(E)**, the secondary cyst wall ist absent due to mechanical damage during tissue processing. **(B,C)** and **(F,G)**: High magnifications of cauliflower-like protrusions (Pr) extended from the primary to the secondary cyst wall, and numerous long dendritic-like filaments **(F)** protruded from both surfaces of the protrusions and the primary wall of the macrocyst. In goats **(G)** the protrusions are more pronounced and the filaments are slightly longer and more numerous than in sheep **(C)**. Within the protrusions, long microtubules (MT) extended longitudinally and transversely from the core of the free protrusions into the ground substance (GS). In both species, coarse and electron-dense granules (ED) of nearly spherical in shape were observed at the base and margin of the projections and in the ground substance. **(D)** and **(H)**: Ultrastructure findings of the bradyzoites of *S. gigantea* and *S. caprafelis moulei*, respectively. They exhibited a bilayered pellicle (Pe), slightely thickened at the anterior end of the banana-shaped bradyzoites and numerous small rod-shaped micronemes (Mn) with rounded ends at the anterior part (Co = conoid). Beneath the micronemes are about 8–12 irregularly shaped rhoptries (Rh), as well as some elongated tubular mitochondria and a relatively small number of amylopectin granules (AG). The cell nucleus (N) is located at the posterior end of the cell body.

The *S. gigantea* and *S. caprafelis moulei* outer secondary cyst wall (PAS-positive in lightmicroscopy) consisted of a thick, fibrillar, collagenous layer, whereas the inner primary cyst wall had a homogeneous appearance. Between the secondary and primary cyst walls, was a layer of the degenerated myofibril remnants consisting of numerous vacuoles of various host cell remnants. The average thickness of the degenerated layer was 4.5 μm in *S. gigantea* (*n* = 40; Figures 3A,B) and 4.9 μm in *S. caprafelis moulei* (*n* = 38; Figures 3E,F). Statistically, no significat difference (*p* < 0.05) was observed between both species (Figure 2K). In addition, the primary macrocyst wall formed cauliflower-, trapezoid- or mushroom resembling protrusions with an average width of 2.4 μm, and a height of 3.1 μm (*n* = 27, *S. gigantea*; Figure 3B). *S. caprafelis moulei* also had similar protrusion features but was slightly larger with an average width of 3.5 and height of 3.4 (*n* = 25; Figures 2I,J, 3F). Statistical analysis revealed a high significant difference (*p* < 0.001) in the width of villar protrusions and a significant difference (*p* < 0.05) in their hight between *S. gigantea* and *S. caprafelis moulei* (Figures 2I,J).

In both species, numerous dendritic-resembling filaments extended from the primary cyst-wall surface and from available surfaces of some protrusions. These filaments were significantly longer and more numerous in goats than in sheep: (Figure 3C in sheep and Figure 3G in goats)–this is the only clear morphological difference between *S. gigantea* and *S. caprafelis moulei*. Moreover, within the cauliflower-resembling protrusions, long microtubules extended longitudinally transversely from the core of the free protrusions into the ground substance of the protrusions (*S. gigantea*: Figures 3B,C; *S. caprafelis moulei*: Figures 3F,G). Coarse, spherical, electron-dense granules occurred at the base and margin of these projections and in the ground substance of the protrusions (Figures 3C,G; *S. gigantea* and *S. caprafelis moulei*, respectively). Furthermore, no marked differences in the ultrastructure of the bradyzoites were detected between these two closely related sister species.

In this study, the fine structure of *S. gigantea* cyst wall (Figures 3A–C) resembled that of the same species described previously [Figure 8.2 (C) in (1)], and the cyst wall of *S. caprafelis moulei* resembled that described briefly in previous studies [compare Figures 1–2 in (17)] with our (Figures 3E–G). In both species, the ground substance had a homogenous appearance: it formed a 3–4 μm thick septa boarder, enclosing the bradyzoites. Immediately below the primary cyst wall, some bradyzoites were visible in the ground substance. Banana-shaped bradyzoites contained a bilayered pellicle, slightly thickened at the anterior end, with a large number of small, rod-shaped micronemes” tiny, apical (23), cigar-shaped organelles involved in host cell recognition and adhesion„ (23). At the anterior end of the bradyzoites, was a rounded end called a “conoid.” This is an intricate structure of spirally arranged microtubules in the apical complex of Apicomplexa that plays a role in host cell entry (*cf.* 23). Behind the micronemes, were approximately 8–12 irregular, varying rhoptries, along with a small amount of elongated, tubular mitochondria and amylopectin granules. Rhoptries, micronemes and electron-dense granules have previously been described as specialized secretory organelles from Apicomplexa parasites (23). The bradyzoites nucleus is mainly located at the posterior end of the body (Figure 3D in sheep, and Figure 3H in goats).

### 3.4. DNA-analysis

#### 3.4.1. Molecular characterization of the 18S rDNA and sequence comparisons

Molecularly, macrocysts from five sheep and one goat were analyzed for differences in their 18S rDNA and 28S rDNA genes. Six of the seven cysts obtained from sheep (numbers: 15a, 15b, 17a, 18, 19 and 20) yielded the same 18S rDNA sequence in a multiple alignment, which was between 82.2 and 89.1% identical to the 18S rDNA genes of *S. gigantea*, *S. medusiformis* and *S. caprafelis moulei* (Tables 1, 2). Macrocyst number 17b presented the highest coresponding sequence rate with three species: 95.6, 98.7 and 95.6%, respectively. The goat-derived cyst number 22 showed between 91.4–93.5% matching sequence to the reference species. Although cysts 17b and 22 showed greater morphological similarities to *S. gigantea, S. medusiformis*, and *S. caprafelis moulei*, they were only 85.9% identical to each other in a pairwise alignment, indicating that the eight macrocysts were derived from three different species. In a phylogenetic tree, cyst 17b was most closely related to *S. medusiformis*, whereas cyst 22 had the fartherest relation to any of the species (A in Tables 1, 2).

**Table 1:** Sequence identity of macrocysts from sheep and goat to *Sarcocystis* spp.

Species	Gene	Accession	Sequence identity (%)							
			15a	15b	17a	17b	18	19	20	22
*S. gigantea*	18S rDNA	MK420020.1	85,2	86,1	86,0	95,6	83,5	89,1	85,5	93,5
	28S rDNA	MK420025.1	99,8	99,9	99,7	94,7	99,5	99,6	99,7	96,8
*S. medusiformis*	18S rDNA	MK420021.1	83,3	85,1	85,2	98,7	82,2	87,7	84,1	91,4
	28S rDNA	MK420026.1	95,0	95,3	95,1	99,3	95,0	95,1	95,0	94,3
*S. caprafelis moulei*	18S rDNA	L76473.1	84,7	86,0	85,8	95,6	83,1	88,7	85,0	92,9
	28S rDNA	AF012884.1	97,1	97,3	97,1	94,3	97,0	97,0	97,0	98,7

Sequence identity (%) of 3 isolated macroscopic Sarcocystis species with nucleotide sequences in the NCBI neucleotide databases of sheep and goats.

**Table 2:** The phylogentic tree of *Sarcocystis* spp. from sheep and goats based on18S rRNA and 28S rRNA genes.

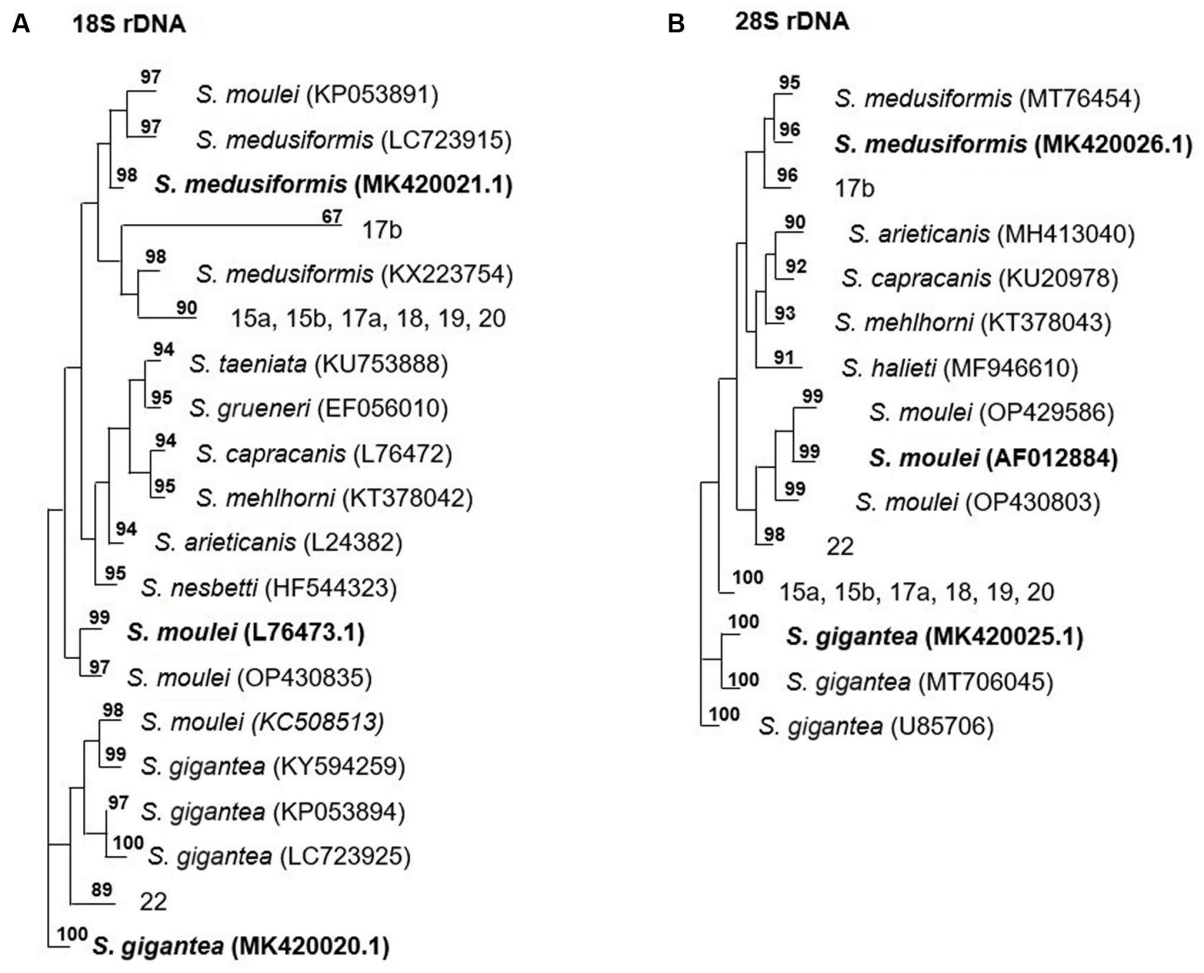

The percentage of replicate trees in which the associated taexa were clustered together in the bootstrap test (1,000 replicates) is indicated next to the branches. Genbank accession numbers of the sequences are indicated after the taxon names.

An NCBI nucleotide blast of the cyst sequences revealed, that the most identical sequence to cyst 17b (accession number MK420021.1), was the reference sequences used in this study for *S. medusiformis* and *S. caprafelis moulei* isolate Sarco 3 MKH 18S ribosomal RNA gene (KP053891.1) both at 99.65%. Cyst 22 was most closely matched the small subunit ribosomal RNA gene of *S. gigantea* isolate 2 (MK045326.1) with 89% sequence identity, while the six other cysts were very similar to an unknown *Sarcocystis* species HS-2009a isolate 1SO 18S ribosomal RNA gene (GQ131808.1) with 99.61% identity.

At the molecular level, using the 18S rRNA sequences, *S. gigantea* showed to be genetically related to *S. medusiformis* in sheep and *S. caprafelis moulei* in goats. The *S. gigantea* sequence was 85.1% identical to *S. medusiformis* and 80.4% identical to *S. caprafelis moulei*. In addition, *S. medusiformis* 85.9% identical to *S. caprafelis moulei*.

#### 3.4.2. Molecular characterization of the 28S rRNA and sequence comparisons

Molecular characterization of the 28S rDNA showed that the same six macrocysts from sheep, as in the 18S rDNA analysis were highly similar in sequence and were closely related to *S. gigantea* with 99.5 to 99.9% sequence identity, followed by *S. caprafelis moulei* with 97.0 to 97.3% and *S. medusiformis* with 95.0 to 95.3% (Table 1). In contrast to the 18S rRNA, the 28S rRNA of 17b cyst sequence was less similar to *S. gigantea* (94.7%) and *S. caprafelis moulei* (94.3%), whereas it showed high similarity to *S. medusiformis,* with 99.3% sequence identity. The 28S rRNA of cyst 22 was closest to that of *S. caprafelis moulei* with 98.7%. Consistent with the 28S rRNA data of all three species, macroysts 17b and 22 had 94.61% sequence identity.

Compared with the 18S rRNA sequence, the 28S rRNA sequence of *S. gigantea* showed closer genetic relationship to *S. medusiformis* in sheep and *S. caprafelis moulei* in goats; this sequence shared an identity of 95.4 and 97.3%, respectively. Furthermore, the new sequence of *S. medusiformis* shared an identity of 94.6% with those of *S. caprafelis moulei*.

The fact that all three randomized cyst groups have a tendency to be most identical to one of the three reference species (A in Table 2) increases the probability of the cysts originating from three different species. A nucleotide blast of the cyst sequences enhanced the pattern of the phylogenetic tree (B in Table 2), as it resulted in the three reference Accession numbers for the 28S rRNA genes used in this study.

## 4. Discussion

*Sarcocystis* species is a parasitic protozoan with a worldwide distribution, found in a variety of mammals and birds, particularly common in domestic animals. *S. gigantea* and *S. medusiformis* in sheep and *S. caprafelis moulei* in goats, which are transmitted by felids, produce macrocysts and are non-pathogenic, although they can cause poor meat quality and economic losses (24, 25). In the present study, the identification of *Sarcocystis* species is based on host specificity, cyst morphology, cyst wall and bradyzoites ultrastructural findings, and molecular characterization.

Microscopic and ultrastructural findings revealed no significant differences between *S. gigantea* and *S. caprafelis moulei* in terms of the size of macrocysts, bradyzoites, and internal compartments, as well as in the cyst wall and degeneration layer thickness. Moreover, no difference in the ultrastructurale features of the brazyzoites were observed between these two closely related species. However, both species contained numerous, dendritic resembling filaments, extending from the primary cyst wall surface and from free surfaces of some protrusions. The only observable difference between the two species is the dendritic-like filaments, which are slightly longer and more numerous in goats than in sheep: (Figure 3C in sheep and Figure 3G in goats). Thus, it is difficult to distinguish these two morphologically related sister species based on ultrastructural findings alone.

Application of molecular methods showed that our DNA sequences are related to *S. gigantea* and *S. medusiformis* in sheep and *S. caprafelis moulei* in goats. In recent years, DNA analysis with high sensitivity and specificity have been used for the identification of *Sarcocystis* species. Based on previous studies, 18S rRNA and/or 28S rRNA genes have been implemented as useful genetic markers for identification and differentiation of *Sarcocystis* in sheep (6, 12, 14, 26) and goats (12, 26).

Analysis of 28S rRNA sequences revealed that *S. gigantea* is closely related to *S. medusiformis* (95.4%) and *S. caprafelis moulei* (97.3%). In addition, our newly discovered sequence of *S. medusiformis* in sheep matched that of *S. caprafelis moulei* in goats 94.6%. Our DNA sequencing indicates that 28S rRNA sequence appear to be more suitable for identifying these species than 18S rRNA sequence.

Macroscopic sarcocystosis prevalency was reported to be 4.1% (13) in domestic sheep in Iraq, 29.3% (27), 3.3% (14) in domestic sheep in Iran and 33.6% [1991–1992; (11)] in domestic goats in Iraq and 16.6% [2004; (16)] in goats in Iran. Encouragingly, the present study showed that these infection rates decreased significantly to only 0.01% (20/141, 260) in domestic sheep and 0.02% (9/37, 399) in domestic goats during the winter season 2021–2022. Our results are confirmed by a previous study in Sulaimany province, where no macroscopic sarcocystosis was found in sheep (0.0%) in a relatively small sample only 130 tissue samples (28). In contrast, the prevelance of macroscopic sarcocystosis was remarkably higher in goats (33.6%) infected, during the winter season 1991–1992 (11), and in goats in Egypt 35.5% (15) with *S. caprafelis moulei* and originating from the same region: a slaughterhouse in Sulaimany province.

Moreover, our infection rate with *S. gigantea* in sheep (0.01%) was also remarkably lower than in sheep slaughtered in Iran in 2011 [29.3%; (28)], Romania in 2007 [26.3%, (7)], and Egypt in 2022 [42.7%; (15)].

This finding could implicate a potential improvement in animal husbandry, reflected in the very low prevalence of macroscopic sarcocystosis. An improvement in animal husbandry could have a positive impact on the general health status of the population. We believe that this improvement is due to several essential factors: (i) Establishment of new, well-organized slaughterhouses with crematoria for incinerating infected carcasses. This plays an important role in breaking the *Sarcocystis* life cycle by preventing definitive hosts from becoming infected or re-infected with *Sarcocystis* and further spreading the sporocysts. (ii) Control of stray dogs and cats, that may serve as definitive hosts by transmitting parasites to certain animal shelters or pet owners. (iii) Control of movement and transport of livestock across Kurdistan Region borders. (iv) Meat consumption of young animals (1–2 years old) due to the economic progress achieved in Iraq in the past 20 years in terms of individual income, where macroscopic sarcocystosis takes a relatively long time to develop in the muscle tissue of their intermediate hosts. (v) Television and social media, to which the Veterinary Office and the Food Control Office turn, are known to play an important role in encouraging people to improve their general knowledge about the handling and consumption of meat.

The successful application of hygienic and eradication procedures in northern Iraq Kurdistan livestock may encourage neighboring countries to adopt a similar strategy.

## 5. Conclusion

To our knowledge, this study is the first: (i) to reveal and describe in detail the macrocyst wall ultrastructural characteristics of *S. caprafelis moulei* in goats and compare them with those of *S. gigantea* in sheep. (ii) In which *S. medusiformis* has been isolated from naturally infected sheep in Iraq. (iii) To investigate the influence of an appropriate hygiene strategy in animal husbandry on the prevalence of sarcocystosis in domestic animals.

## Data availability statement

The data presented in the study are deposited in the GenBank NCBI nucleotide databases under the accession numbers: MK420020.1, MK420025.1, MK420021.1, MK420026.1, L76473.1, AF012884.1, KP053891.1, MK045326.1, and GQ131808.1.

## Ethics statement

Ethical approval was not required for the study involving animals in accordance with the local legislation and institutional requirements because it involved the use of animal tissues obtained post mortem from animals sacrificed for non-scientific purposes. This study was conducted under the supervision of the Central Veterinary Office in Sulaimany, Kurdistan Region of Iraq, and within the framework of the regional animal protection law.

## Author contributions

SN: data curation and inspection. NH: DNA-analysis, wrote a part of manuscript with contributions with MB. NP: DNA-extraction and software. TB: electron microscopy. NF-S: interpreted data. AG: interpreted data and software. WN: interpred and analyzed data. JV: funding acquisition, interpreted data, and software. MB: data curation, electron microscopy, software, writing-original draft, and supervised this research.

## Footnotes

^1^ https://srvweb.microsynth.ch/

^2^ https://blast.ncbi.nlm.nih.gov/Blast.cgi
