# Supplementary material for: Morphological, ultrastructural, genetic characteristics and remarkably low prevalence of macroscopic Sarcocystis species isolated from sheep and goats in Kurdistan region, Iraq
Source: Front Vet Sci. 2023 Sep 28;10:1225796. doi: 10.3389/fvets.2023.1225796 (PMC10569315; doi:10.3389/fvets.2023.1225796)
Supplement: Supplementary file 1 [file Table_1.docx]

| **Supplement 01.** Slaughtering data of sheep and goats in six Abattoirs in the Kurdistan Region Northern, Iraq. Data in brackets are the numbers of infected animals. |
| --- |
|  |

| **Abattoir** | **Animals** | | | **Months** | | | | | | | |  |
| --- | --- | --- | --- | --- | --- | --- | --- | --- | --- | --- | --- | --- |
|  |  |  |  | **Sep. 21/+ve** | **Oct.21/+ve** | **Nov.21/+ve** | **Dec.21/+ve** | **Jan.22/+ve** | **Feb.22/+ve** | **Mar.22/+ve** | **Total** | |
|  |  |  |  |  |  |  |  |  |  |  |  | |
| **Slemany** | **Sheep** | | **M** | 11572 | 12528 | 15131 (1) | 1892 | 13370 | 13821 | 19023 | **87337** | |
|  |  |  | **F** | 4851 | 5033 (2) | 4735 (2) | 3701(1) | 6001 (2) | 7893 | 8625 | **40839** | |
|  | **Goats** | | **M** | 11182 | 1993 | 1075 | 4009 | 3241 | 1856 | 2299 | **25655** | |
|  |  |  | **F** | 421 | 691 (2) | 728 | 814 (2) | 611 | 247 | 382 | **3894** | |
| **Piramag-**  **roon** | **Sheep** | | **M** | 245 | 151 | 251 (1) | 57 | 223 | 179 | 356 | **1462** | |
|  |  |  | **F** | 0 | 0 | 0 | 0 | 0 | 0 | 0 | **0** | |
|  | **Goats** | | **M** | 251 | 139 | 85 | 44 | 83 | 45 | 35 | **682** | |
|  |  |  | **F** | 0 | 0 | 0 | 0 | 0 | 0 | 0 | **0** | |
| **Khalakan** | **Sheep** | | **M** | 340 | 473 | 485 | 462 | 505 | 468 | 509 | **3242** | |
|  |  |  | **F** | 156 | 78 | 215 (1) | 29 | 269 | 151 | 230 | **1128** | |
|  | **Goats** | | **M** | 130 | 155 | 239 | 151 | 181 | 107 | 286 | **1249** | |
|  |  |  | **F** | 34 | 90 | 65 | 94 (2) | 116 | 78 | 67 | **544** | |
| **Darband-**  **ixan** | **Sheep** | | **M** | 220 | 250 | 260 (1) | 125 | 320 | 170 | 200 | **1545** | |
|  |  |  | **F** | 60 | 40 | 40 | 20 (1) | 150 | 70 | 80 | **460** | |
|  | **Goats** | | **M** | 230 | 240 | 250 | 130 | 250 | 150 | 170 | **1420** | |
|  |  |  | **F** | 50 | 50 | 50 | 20 | 150 | 50 | 60 | **430** | |
| **Takya** | **Sheep** | | **M** | 530 | 500 | 480 (1) | 460 (1) | 450 | 455 | 470 | **3345** | |
|  |  |  | **F** | 20 | 20 (1) | 20 (1) | 20 | 50 (2) | 35 | 30 | **195** | |
|  | **Goats** | | **M** | 160 | 140 | 140 | 130 | 90 | 90 | 100 | **850** | |
|  |  |  | **F** | 10 | 10 | 0 | 0 | 50 | 10 | 10 | **90** | |
| **Soran** | **Sheep** | | **M** | 152 | 130 | 130 (1) | 117 (1) | 406 | 336 | 436 | **1707** | |
|  |  |  | **F** | 0 | 0 | 0 | 0 | 0 | 0 | 0 | **0** | |
|  | **Goats** | | **M** | 541 | 504 | 480 | 289 | 234 (1) | 264 (2) | 273 | **2585** | |
|  |  |  | **F** | 0 | 0 | 0 | 0 | 0 | 0 | 0 | **0** | |
| **Total** | | | | **31155** | **23215 (5)** | **24859 (9)** | **12564 (8)** | **26750 (5)** | **26475 (2)** | **33641** | **178659** | |
|  |  | Female | Male | Total | +ve Female | +ve Male |  |  |  |  |  | |
| **Sheep** |  | 42622 | 98638 | 141260 | 13 | 7 |  |  |  |  |  | |
| **Goats** |  | 4958 | 32441 | 37399 | 6 | 3 |  |  |  |  |  | |
|  |  |  |  |  |  |  |  |  |  |  |  | |
