# Supplementary material for: Morphological, ultrastructural, genetic characteristics and remarkably low prevalence of macroscopic Sarcocystis species isolated from sheep and goats in Kurdistan region, Iraq
Source: Front Vet Sci. 2023 Sep 28;10:1225796. doi: 10.3389/fvets.2023.1225796 (PMC10569315; doi:10.3389/fvets.2023.1225796)
Supplement: Supplementary file 2 [file Table_2.docx]

| **Species** | **Macrocysts length x width [mm]** | **Total wall thickness [µm]** | **Wall thickness [µm]** | | **Area compart-ments [µm²]** | **Bradyzoites length x width [µm]** | **Degenerated layer thickness [µm]** | **Villar protrusions width x height [µm]** |
| --- | --- | --- | --- | --- | --- | --- | --- | --- |
|  |  |  | **Inner primary wall** | **Outer secondary wall** |  |  |  |  |
| ***S. gigantea*** | 6.7±3.1 x 2.9±1.3  range = 3.4-17.1 x 1.0-6.8  (n=32) | 18.9±7.5  range = 7.1-29.1  (n=25) | 7.3±1.9 range = 4.2-10.7 (n=25) | 11.6±6.0 range 3.0-20.4 (n=25) | 1065±695  range = 234-2886  (n=68) | 12.3±3.8 x 2.6±0.5  range = 7-25 x 2-4  (n=48) | 4.5±1.7  Range 1.3-8.4 (n= 40) | 2.4±1.4 x 3.1±1.6  range 1.2-5.6 x 1.0-8.5 (n= 28) |
| ***S. caprafelis moulei*** | 6.3±2.8 x 4.0±1.9  range = 3.0-13.8 x 2.0-10.2  (n=12) | 15.3±1.7  range = 12.9-18.0  (n=7) | 7.3±0.4  range = 6.5-8.0 (n=7) | 8.0±1.6  range = 6.0-10.4 (n=7) | 917±626  range = 317-3093  (n=94) | 13.9±1.1 x 4.4±0.7  range = 11-16 x 3-6  (n=30) | 4.9±1.9  range 1.7- 10.2  (n = 40) | 3.5±1.1 x 3.4±0.9  range 2.12-5.93 x 2.0-5.8  (n=23) |

**Supplement 2**. Shows dimensions of macrocysts, area compartments, bradyzoites, and villous protrusions, as well as the thickness of macrocyst walls and degenerated layers.
