# Supplementary material for: Morphological, ultrastructural, genetic characteristics and remarkably low prevalence of macroscopic Sarcocystis species isolated from sheep and goats in Kurdistan region, Iraq
Source: Front Vet Sci. 2023 Sep 28;10:1225796. doi: 10.3389/fvets.2023.1225796 (PMC10569315; doi:10.3389/fvets.2023.1225796)
Supplement: Supplementary file 3 [file Table_3.docx]

**Supplement 03.** The sequencing data of our material are deposited in NCBI.

**Sequences Accession numbers**

**28S rRNA**

SUB13473962 Seq1         OR048077
SUB13473962 Seq2         OR048078
SUB13473962 Seq3         OR048079
SUB13473962 Seq4         OR048080
SUB13473962 Seq5         OR048081
SUB13473962 Seq6         OR048082
SUB13473962 Seq7         OR048083
SUB13473962 Seq8         OR048084
SUB13473962 Seq9         OR048085
SUB13473962 Seq10       OR048086
SUB13473962 Seq11       OR048087
SUB13473962 Seq12       OR048088
SUB13473962 Seq13       OR048089
SUB13473962 Seq14       OR048090
SUB13473962 Seq15       OR048091
SUB13473962 Seq16       OR048092

**18S rRNA**

SUB13473577 Seq1         OR052228
SUB13473577 Seq2         OR052229
SUB13473577 Seq3         OR052230
SUB13473577 Seq4         OR052231
SUB13473577 Seq5         OR052232
SUB13473577 Seq6         OR052233
SUB13473577 Seq7         OR052234
SUB13473577 Seq8         OR052235
SUB13473577 Seq9         OR052236
SUB13473577 Seq10       OR052237
SUB13473577 Seq11       OR052238
SUB13473577 Seq12       OR052239
SUB13473577 Seq13       OR052240
SUB13473577 Seq14       OR052241
SUB13473577 Seq15       OR052242
SUB13473577 Seq16       OR052243
