# Supplementary material for: Morphological, ultrastructural, genetic characteristics and remarkably low prevalence of macroscopic Sarcocystis species isolated from sheep and goats in Kurdistan region, Iraq
Source: Front Vet Sci. 2023 Sep 28;10:1225796. doi: 10.3389/fvets.2023.1225796 (PMC10569315; doi:10.3389/fvets.2023.1225796)
Supplement: Supplementary file 4 [file Data_Sheet_1.PDF]

Extraction of genomic DNA

1. Add 250µl of lysis buffer per sample (divided).
2. Add 20µl of Proteinase K (600U/ml = 30U/mg) and vortex.
3. Incubate overnight at 55°C in a thermomixer at 600rpm.
4. Melt the tip of a Pasteur pipette.
5. Centrifuge the digestion product at 14000 rpm for 8 minutes.
6. Transfer the supernatant to 0.5 ml of 100% ethanol (-20°C).
7. DNA will precipitate and form threads.
8. Use the melted Pasteur pipette tip to wind up the DNA threads, then dip the tip in 375µl of 70% ethanol (-20°C).
9. Invert the Pasteur pipette tip with DNA threads and let it air dry on a rack for 10 minutes.
10. Break off the Pasteur pipette tip with DNA threads in an Eppendorf tube containing 75µl of Milli-Q water.
11. Allow the DNA to dissolve overnight at 42°C.
12. Invert the Eppendorf tube.

    The DNA is now dissolved and ready for further experiments.

Lysis buffer:

Stable at room temperature for approximately 2-3 months

10ml 1M Tris pH 8

5ml 0.5M EDTA pH 8

1ml 5M NaCl

5ml 10% SDS

24ml Milli-Q water
